# Prevalence of *Giardia* infection in households of *Giardia* cases and risk factors for household transmission

**DOI:** 10.1186/s12879-017-2586-3

**Published:** 2017-07-11

**Authors:** Alison Waldram, Roberto Vivancos, Catherine Hartley, Kenneth Lamden

**Affiliations:** 1 Field Epidemiology Service, Public Health England, Liverpool, UK; 2 Field Epidemiology Training Programme, Public Health England, London, UK; 3 European Programme for Intervention Epidemiology Training, European Centre for Disease Prevention and Control, Stockholm, Sweden; 4 NIHR Health Protection Research Unit in Gastrointestinal Infections, University of Liverpool, Liverpool, UK; 5 NIHR Health Protection Research Unit in Emerging & Zoonotic Infections, University of Liverpool, Liverpool, UK; 6 Department of Infection Biology, Institute of Infection and Global Health, University of Liverpool, Liverpool, UK; 7 Cumbria and Lancashire Health Protection Team, Public Health England, Preston, UK

**Keywords:** *Giardia*, Household, Transmission, Assemblage

## Abstract

**Background:**

*Giardia* is a leading but neglected cause of infectious gastroenteritis worldwide and is treatable. There is a substantial burden of undetected *Giardia* in the UK and for every one case of *Giardia* reported to national surveillance there are 14 cases in the community. We aimed to ascertain the prevalence of, and risk factors associated with secondary household *Giardia* infections to assess the burden of infection and inform control measures.

**Methods:**

We identified all giardiasis cases notified in nine local authorities in Lancashire between June 2014 and June 2015, and invited their household contacts to submit faecal specimens for *Giardia* testing and complete a risk factor questionnaire. We estimated the proportion of households with additional *Giardia* infection. We compared household risk factors between households with and without additional *Giardia* using Fisher’s exact test. We used multivariable logistic regression to identify independent risk factors for additional *Giardia* infections.

**Results:**

We identified additional *Giardia* infections in 30% (27/91) of included households. A total of 41 infections were found from 212 household members, of which 37 were asymptomatic. The majority of infections were assemblage B (57%) but there were also a high number of mixed infections (20%). Risk factors significantly associated with additional household infections were; having children under 5 years in the household (odds ratio 42; 95% confidence intervals 10–178) and the presence of gastrointestinal illness in the household before the onset of the index case (odds ratio 9; 95% confidence intervals 1.5–48).

**Conclusions:**

Our finding of a high household prevalence of asymptomatic infection has raised the public health question of whether treatment of asymptomatic household contacts may be justified in preventing *Giardia* re-infection of the index case or in preventing secondary cases and household clusters. We recommend the communication of this risk in household contacts of *Giardia* and reinforcement of standard hygiene controls.

## Background

*Giardia* is a leading but neglected cause of infectious gastroenteritis worldwide [1] and is treatable. The flagellated protozoan, *Giardia lamblia* (syn. *G. duodenalis* and *G. intestinalis*) comprises eight genetic “assemblages” (A-H) with only A and B affecting humans. Assemblages A and B can also infect pets, livestock and wild animals showing the potential for zoonotic transmission [2]. The reported prevalence of *Giardia* in human populations is 4–43% and 1–7% in low and high income countries respectively [3, 4]. Prevalence in the UK has been reported as 1.3% in asymptomatic children [5], 1.4% in a general practitioner population and 0.9% in the general population [6]. Between 3000 and 4000 cases are reported annually in England and Wales [7]. There is a substantial burden of undetected *Giardia* in the UK and for every one case of *Giardia* reported to national surveillance there are 14 cases in the community [8]. Corresponding ratios for *Campylobacter*, *Cryptosporidium* and *Salmonella* are 9.3, 8.2 and 4.7 respectively. The incidence of *Giardia* in Northwest England increased four-fold following the introduction of the enzyme linked-immunosorbent assay for the detection of parasite antigens in stools [9]. This test has greater sensitivity than microscopy [10] and was applied universally to all stool samples, i.e. no testing criteria were applied, indicating that the majority of cases did not have the commonly accepted exposures for *Giardia* cases.

*Giardia* transmission occurs through the ingestion of the infective cyst stage shed in human or animal faeces. The cyst may be present in faecally contaminated water, food or fomites. The clinical disease (giardiasis) typically includes diarrhoea, flatulence, abdominal pain and bloating [11] and weight loss due to malabsorption [12]. Some infections are relapsing due to re-infection from an ongoing source, possibly an asymptomatic household member, or because they are refractory to metronidazole therapy [13]. The clinical picture is altered in high prevalence countries due to partial immunity from repeated exposure. Chronic infection in children may cause failure to thrive [14].

Estimates of the proportion of *Giardia* infections that are asymptomatic, but which have the potential for transmission of *Giardia* cysts, varies considerably from 5 to 15% [15] to 76% [16]. Without treatment infectivity can continue for months, potentially causing household clusters or outbreaks. There is no evidence based guidance on when to treat asymptomatic infection and when it may curtail transmission, and asymptomatic carriage is generally not treated [17, 18]. However expert opinion suggests treatment of asymptomatic *Giardia* infection in certain circumstances, for example food handlers, day care nurseries and recurrent infection in a household [19, 20].

The aim of this study was to estimate the prevalence of *Giardia* infection in households of index cases of giardiasis and to identify characteristics of households with more than one case of giardiasis. The study investigated infected rather than diseased cases (defined as symptomatic and therefore more likely to be reported) to provide a more comprehensive insight into transmission dynamics and risk factors within Northwest England households.

## Methods

### Prevalence survey

An observational study was conducted to estimate the prevalence of *Giardia* infection in households of confirmed *Giardia* cases in nine local authorities in Lancashire between June 2014 and June 2015. *Giardia* cases were identified by the detection of *Giardia* antigen in a stool specimen using a faecal antigen enzyme immunoassay (EIA) method as previously described [9, 10], by the three participating hospital laboratories in Lancaster, Blackburn and Preston. All positive specimens were further characterised by assemblage, details of the methods are described [21].

Households were invited to participate by an environmental health officer during their routine public health investigation. All household members were asked to provide a stool sample. A household member was defined pragmatically as a person who lived in the same residence as the case for at least two nights per week in the month prior to diagnosis or had household equivalent contact for example in a care home or university residence. All stool samples were tested using the same EIA method as the index cases. Index cases were excluded if they lived in a single person household.

The prevalence of additional *Giardia* infection was measured by dividing the number of households that had at least one case of *Giardia* infection in addition to an index case by the total number of households included in the study. The *Giardi*a prevalence amongst household contacts was also estimated by dividing the total number of additional infections detected by the total number of household contacts.

The sample size was based on an estimated additional *Giardia* household prevalence of 10% (local surveillance data showed that a second symptomatic case of *Giardia* was reported in 7% of households plus an additional 3% estimated for asymptomatic individuals). The significance level used was 0.05 (corresponding to 95% confidence intervals) with a confidence width of 0.05 with an estimated a response rate of 80% the sample size was 130 households.

### Cross-sectional analysis

We compared households with and without secondary infection to determine the characteristics of households with secondary infections. A “case” household was defined as a household with at least one additional *Giardia* infection in a household member. A “control” household had no additional *Giardia* infections. We compared household characteristics between “case” and “control” households using univariable analyses to calculate odds ratios (OR) and *p* values using Fisher’s exact test. The characteristics were identified from the index case study questionnaire and additional assemblage information. We used multivariable logistic regression to identify independent risk factors for additional *Giardia* infections. All risk factors that had a *p* value less than 0.2 were considered in a multivariable analysis. The final model included risk factors that were significantly associated with additional *Giardia* infections i.e. *p* value <0.05.

Questionnaire information was entered using EpiData and data was analysed using Stata v12 (StatCorps). The date of onset for cases was estimated from the in-house Public Health England case management system if not available from the questionnaire. The dataset was checked for accuracy using Stata to clean the data and check for impossible values and missing data items were sought via environmental health officers or the in-house case management system.

## Results

### Prevalence survey

**Fig. 1:**
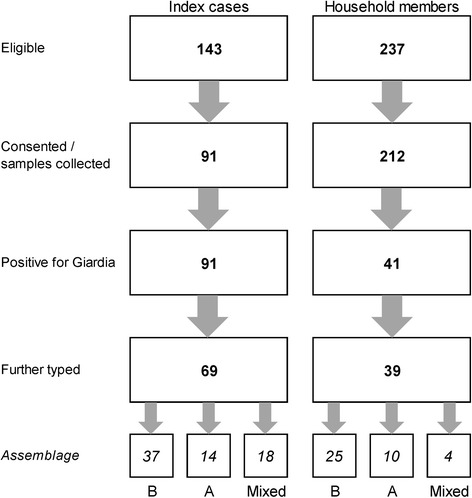
Numbers of participants included in the study including details of positivity and further typing

The number of index *Giardia* cases identified was 186. Of these 17 were excluded as a single person household and 26 could not be contacted, resulting in 143 eligible index cases (Fig. 1). Of these, 91 households participated giving a response rate of 64%. There were no significant differences between households that participated and those that didn’t for household size, age or sex of the index case. Within the 91 study households there were 237 household members of whom 212 (89%) provided a stool sample (Fig. 1).

At least one additional case of *Giardia* infection was detected in 27 households, giving a household prevalence of 30% (27/91) (95% confidence intervals (CI): 20–39%). *Giardia* was detected in 41/212 (19%) of household members of whom 37 (90%) were asymptomatic, giving a prevalence of asymptomatic infection of 17% (37/212) (95% CI: 12–23%) and a prevalence of undetected symptomatic infection of 2% (4/212) (95% CI: 0.06–3.7%). The age groups most affected by asymptomatic infection were 0–4 and 5–9, with 51% and 35% of household members being affected, respectively (Table 1). The number of additional *Giardia* positive individuals in a single household ranged from one to five.

**Table 1:** Number of household members by asymptomatic infection status and percentage of asymptomatic infection in household members

Age group of household members	Asymptomatic infections in household members		Household members with no infection		Total household members^a^		Percentage of asymptomatic infection in household members
	Number	*%*	Number	*%*	Number	*%*	
0–4	19	*51*	18	*12*	37	*19*	51.4
5–9	8	*22*	15	*10*	23	*12*	34.8
10–19	1	*3*	33	*21*	34	*18*	2.9
20–29	3	*8*	13	*8*	16	*8*	18.8
30–39	4	*11*	27	*18*	31	*16*	12.9
40–49	1	*3*	17	*11*	18	*9*	5.6
50+	1	*3*	31	*20*	32	*17*	3.1
Total	37	*100*	154	*100*	191	*100*	19.4

^a^4 symptomatic household members and 17 household members with no infection and no available age were excluded

### Cross-sectional analysis

The univariable analyses for risk factors when comparing households with additional *Giardia* infection against those without additional *Giardia* infection can be seen in Table 2.

**Table 2:** Univariable analyses comparing households with additional *Giardia* infection to those without

Risk factor / characteristic	Households **with** additional *Giardia* infection			Households **without** additional *Giardia* infection			Odds Ratio	95% CI	*P* value
	Total	Exposed	%	Total	Exposed	%			
Children (<5) in household	27	21	*77.8*	64	7	*10.9*	28.50	[7.55–113.54]	0.000
Less than 1 bedroom per person in household	27	16	*59.3*	64	17	*26.6*	4.02	[1.41–11.59]	0.004
Assemblage B in the index case	25	24	*96.0*	43	30	*69.8*	10.40	[1.34–459.95]	0.012
Children in nappies in household	24	16	*66.7*	22	7	*31.8*	4.29	[1.07–17.73]	0.038
Less than 1 toilet per person in household	27	23	*85.2*	64	41	*64.1*	3.23	[0.93–14.24]	0.049
Female index case	27	15	*55.6*	64	21	*32.8*	2.56	[0.92–7.13]	0.060
Cat in household	27	3	*11.1*	61	19	*31.2*	0.28	[0.05–1.10]	0.062
4 or more people in household	27	18	*66.7*	64	28	*43.8*	2.57	[0.92–7.48]	0.066
Involved in changing nappies	20	10	*50.0*	22	5	*22.7*	3.40	[0.76–16.21]	0.107
Assemblage A in the index case	25	8	*32.0*	43	23	*53.5*	0.41	[0.13–1.28]	0.130
Vomiting in index case	26	16	*61.5*	60	26	*43.3*	2.09	[0.74–6.04]	0.160
Index case under 5 years old	27	6	*22.2*	64	6	*9.4*	2.76	[0.65–11.48]	0.171
Illness in household before index case	27	6	*22.2*	59	6	*10.2*	2.52	[0.59–10.53]	0.181
Children that go to nursery in household	24	13	*54.2*	21	7	*33.3*	2.36	[0.61–9.53]	0.231
Dog/cat in household	27	12	*44.4*	64	38	*59.4*	0.55	[0.20–1.49]	0.250
Duration of illness greater than 7 days	26	26	*100.0*	57	53	*93.0*	.	[0.48-.]	0.304
Previous *Giardia* in index case	27	0	*0.0*	60	4	*6.7*	0.00	[0.00–2.10]	0.306
Index case travelled abroad before onset	27	7	*25.9*	61	22	*36.1*	0.62	[0.19–1.85]	0.462
Dog in household	27	12	*44.4*	62	23	*37.1*	1.36	[0.49–3.73]	0.638
Dog in household under 2 years	18	1	*5.6*	41	5	*12.2*	0.42	[0.01–4.28]	0.656
Index case contact with other illness	26	3	*11.5*	56	4	*7.1*	1.70	[0.23–10.85]	0.673
Assemblage AB in the index case	25	7	*28.0*	43	10	*23.3*	1.28	[0.35–4.50]	0.773
Any pet in household	27	16	*59.3*	61	34	*55.7*	1.16	[0.42–3.24]	0.818
White British index case	27	24	*88.9*	64	57	*89.1*	0.98	[0.20–6.38]	1.000
Dog or cat in household under 2 years	20	2	*10.0*	48	7	*14.6*	0.65	[0.06–3.92]	1.000
Index case resident in rural location	27	3	*11.1*	64	6	*9.4*	1.21	[0.18–6.22]	1.000
Diarrhoea in index case	26	25	*96.2*	63	61	*96.8*	0.82	[0.04–50.26]	1.000
Cat in household under 2 years	13	1	*7.7*	35	2	*5.7*	1.38	[0.02–28.53]	1.000
Household is a farm	26	0	*0.0*	58	1	*1.7*	0.00	[0.00-.]	1.000
Household has a garden	26	19	*73.1*	59	42	*71.2*	1.10	[0.36–3.67]	1.000

The highest association with additional household *Giardia* infection was with having a child under 5 years old in the household (OR 29; 95% CI 8–114). There were other risk factors linked to having children; children in nappies in the household (OR 4; 95% CI 1–18), being involved in changing nappies in the household (OR 3; 95% CI 0.8–16), being an index case under 5 years old (OR 3; 95% CI 0.7–11) and children attending nursery in the household (OR 2; 95% CI 0.6–10). Other characteristics associated with additional infection were related to the number of people and ratio to facilities; having less than one bedroom per person in the household (OR 4; 95% CI 1–12), having less than one toilet per person in the household (OR 3; 95% CI 1–14) and having four or more people in the household (OR 3; 95%CI 1–8). No association was found between having additional *Giardia* infection in the household and owning a cat (OR 0.3; 95% CI 0.5–1) and/or dog (OR 0.6: 95% CI 0.4–2). Of the four index cases with previous *Giardia* infection (ranging from 3 to 26 months prior to the current infection), none had any additional infection in the household.

In the multivariable analysis two risk factors remained significantly associated with additional *Giardia* infection in the household; having children under 5 years in the household (OR 42.35; 95% CI 10.09–177.69) and having anyone with gastrointestinal symptoms in the household in the 3 weeks before the index case (OR 8.55; 95% CI 1.51–48.28).

Genetic analysis was able to assign an assemblage to 108/132 (82%) specimens. Of these 62 (57%) were assemblage B, 24 (20%) assemblage A and 22 (20%) were mixed A and B. Three households did not have assemblage typing completed. If the index case in the household had assemblage A, the household was significantly less likely to have any additional infection (OR 0.1; 95% CI 0–0.8). The assemblage of the index case and the assemblage of the household members were concordant in 92% of households (22/24). This included both single assemblage concordance i.e. A and A or B and B (54%; 13/24) and where there was mixed infection and single assemblages present e.g. A/B and A (38%; 9/24).

## Discussion

To our knowledge this is the first study of *Giardia* prevalence amongst household contacts of sporadic *Giardia* cases. During an outbreak of giardiasis in 1977 no additional cases of *Giardia* were found in 23 household contacts of eight index cases [15]. In contrast we found a high prevalence of asymptomatic *Giardia* infection with an additional *Giardia* infection detected in 30% of households and 17% of all household contacts were found to have asymptomatic infection. This high prevalence may fuel transmission in the household and in the community to a greater extent than is currently recognised. Only four additional symptomatic infections were identified however this was a point prevalence and the true rate of symptomatic infection amongst household contacts will be higher and requires evaluation by a longitudinal study of *Giardia* infected households.

Limitations of this study included the small sample size and the single geographical study area. Further studies would determine whether the findings were generalisable more widely in a low *Giardia* prevalence setting taking account of *Giardia* diagnostic methods and environmental and socioeconomic factors, for example household size and type of housing. Although a small study, the sample size was sufficient to provide an estimate of the true prevalence of additional asymptomatic infections. The response rate of 64% was lower than anticipated although it seems unlikely that the *Giardia* prevalence in non-responding households would be substantially different to responding households due to the similarity in demographics and risk factors. A potential limitation of the study - poor compliance with stool sampling by asymptomatic household members – did not occur, with a compliance rate of 89% adding to the strength of the findings.

We found a strong association between the presence of additional *Giardia* cases and having children aged under 5 years in the household. The transmission of *Giardia* has been reported frequently in day care or nursery settings previously [5, 22] and has been shown to be associated with changing nappies [23]. This result adds to the body of evidence that close contact with children, even those without symptoms, can play an important role in the transmission of *Giardia.*

Developing a ‘high risk household’ definition may be useful for the communication of risk and advising households on ways of reducing this risk. Household factors such as a greater concentration of individuals and fewer facilities also increased the risk of transmission. This may be related to a higher risk of transmission from a contaminated toilet area in households with proportionately fewer toilet facilities per household member. A larger study powered to investigate this association is required to confirm this finding.

Assemblage typing found that the majority of infections were assemblage B, which has previously been associated with person to person transmission [21, 22]. There were a high percentage of mixed assemblage infections (20%). The concordance rate of index and secondary household cases has not been previously reported. Two households had discordant assemblages, possibly due to differing sources or a missed mixed infection in the household.

## Conclusions

Our finding of a high household prevalence of asymptomatic infection has raised the public health question of whether treatment of asymptomatic household contacts may be justified in preventing *Giardia* re-infection of the index case or in preventing household clusters. This is particularly pertinent in households containing children under 5 years in which 50% of household members under 5 years had asymptomatic *Giardia* infection. Currently, asymptomatic carriage is generally not treated due to lack of evidence, but treatment seems rational in failed treatment of a case or where there is a household cluster. Evidence of the effectiveness of treating asymptomatic infection in curtailing transmission could lead to the offer of routine testing of household contacts or a pragmatic alternative of offering blind treatment to all household contacts. Wider availability of sensitive PCR diagnostic tests may aid a more targeted approach to contact treatment in the future. As this was a prevalence study it cannot provide evidence on the impact of treating asymptomatic infection. A longitudinal study on a larger population is required before recommending any change in current practice.

## Abbreviations

CI: Confidence interval
EIA: Enzyme immunoassay
HPRU: Health protection research unit
NIHR: National Institute for Health Research
OR: Odds ratio
PHE: Public Health England
